# Direct stimulation of human fibroblasts by nCeO_2_*in vitro* is attenuated with an amorphous silica coating

**DOI:** 10.1186/s12989-016-0134-8

**Published:** 2016-05-04

**Authors:** Donna C. Davidson, Raymond Derk, Xiaoqing He, Todd A. Stueckle, Joel Cohen, Sandra V. Pirela, Philip Demokritou, Yon Rojanasakul, Liying Wang

**Affiliations:** 1 National Institute for Occupational Safety and Health, Health Effects Laboratory Division, 1095 Willowdale Road, Morgantown, WV 26505 USA; 2 Department of Environmental Health, Center for Nanotechnology and Nanotoxicology, Harvard T. H. Chan School of Public Health, Boston, MA USA; 3 Department of Pharmaceutical Sciences and Mary Babb Randolph Cancer Center, West Virginia University, Morgantown, WV USA

**Keywords:** Cerium oxide nanoparticles, Fibrosis, Engineered nanomaterials, Nanotoxicology, *in vitro* dosimetry

## Abstract

**Background:**

Nano-scaled cerium oxide (nCeO_2_) is used in a variety of applications, including use as a fuel additive, catalyst, and polishing agent, yet potential adverse health effects associated with nCeO_2_ exposure remain incompletely understood. Given the increasing utility and demand for engineered nanomaterials (ENMs) such as nCeO_2_, “safety-by-design” approaches are currently being sought, meaning that the physicochemical properties (e.g., size and surface chemistry) of the ENMs are altered in an effort to maximize functionality while minimizing potential toxicity. *In vivo* studies have shown in a rat model that inhaled nCeO_2_ deposited deep in the lung and induced fibrosis. However, little is known about how the physicochemical properties of nCeO_2,_ or the coating of the particles with a material such as amorphous silica (aSiO_2_), may affect the bio-activity of these particles. Thus, we hypothesized that the physicochemical properties of nCeO_2_ may explain its potential to induce fibrogenesis, and that a nano-thin aSiO_2_ coating on nCeO_2_ may counteract that effect.

**Results:**

Primary normal human lung fibroblasts were treated at occupationally relevant doses with nCeO_2_ that was either left uncoated or was coated with aSiO_2_ (amsCeO_2_). Subsequently, fibroblasts were analyzed for known hallmarks of fibrogenesis, including cell proliferation and collagen production, as well as the formation of fibroblastic nodules. The results of this study are consistent with this hypothesis, as we found that nCeO_2_ directly induced significant production of collagen I and increased cell proliferation *in vitro*, while amsCeO_2_ did not. Furthermore, treatment of fibroblasts with nCeO_2_, but not amsCeO_2_, significantly induced the formation of fibroblastic nodules, a clear indicator of fibrogenicity. Such *in vitro* data is consistent with recent *in vivo* observations using the same nCeO_2_ nanoparticles and relevant doses. This effect appeared to be mediated through TGFβ signaling since chemical inhibition of the TGFβ receptor abolished these responses.

**Conclusions:**

These results indicate that differences in the physicochemical properties of nCeO_2_ may alter the fibrogenicity of this material, thus highlighting the potential benefits of “safety-by-design” strategies. In addition, this study provides an efficient *in vitro* method for testing the fibrogenicity of ENMs that strongly correlates with *in vivo* findings.

## Background

Cerium, of the lanthanide metal group, is considered a rare earth metal. Rare earth metals have been reported to induce pneumoconiosis upon occupational exposure, with cerium found to persist in the alveoli and interstitial tissue for decades even after exposure has ceased [1]. Indeed, even the term “cerium pneumoconiosis” has been coined due to the increasing prevalence of such disorders [2, 3]. Use of cerium oxide nanoparticles (nCeO_2_) has been steadily increasing across many industries, as it is an efficient polishing agent for glass, mirrors, and ophthalmic lenses. In addition, it has been utilized as a diesel fuel catalyst to aid in emission reduction, which subsequently leads to its release in the particulate phase of exhaust [4, 5]. Other uses of nCeO_2_ in consumer goods are also in development, including use as an additive in cosmetics and sunscreens based on its UV absorption properties, and within the pharmaceutical industry. Thus, human exposure is inevitable in environmental, consumer, and occupational settings, with inhalation being the primary route of exposure.

Unfortunately, data is somewhat limited regarding human exposure concentrations to nCeO_2_ in either occupational or environmental settings. However, several individual case studies of movie projectionists and photoengravers with regular exposure to nCeO_2_ have noted the bio-persistence of these particles following occupational exposures, with associated lung disease [6, 7]. Considering the increased demand and use of this material, these reports highlight the need to fully characterize potential exposure-induced health effects associated with nCeO_2_ use.

Consistent with this notion, several studies have been conducted in an effort to better understand nCeO_2_-induced effects, with a focus on animal models of disease. These reports have noted the presence of nCeO_2_-induced pulmonary inflammation, cardiovascular dysfunction, and fibrosis, as well as the bio-accumulation of nCeO_2_, in agreement with the observed effects reported in human case studies [8–14]. Fibrosis is characterized by the excessive production of extracellular matrix (ECM) components around damaged lung tissue, which accumulate as the typical wound healing response mediated by fibroblasts aberrantly persists [15]. Under normal circumstances, following inflammation or injury, fibroblasts within the lung interstitium produce ECM proteins, such as collagen and fibronectin, in an effort to repair the damaged connective tissue. However, if left unchecked, fibrotic scarring can occur. Unfortunately, very few, if any, therapeutic strategies for the management of fibrosis currently exist, underlying the importance of devising strategies to limit the chances of developing fibrosis in occupational, consumer, and environmental settings.

Given the increasing utility and demand for engineered nanomaterials (ENMs) such as nCeO_2_, “safety-by-design” approaches are currently being sought in an effort to maximize the functionality of ENMs while minimizing their potential toxicity and adverse health effects. One such strategy is the use of surface coatings, such as a nano-thin amorphous silica (aSiO_2_) coating, that would provide a biologically inert shell while retaining the desired core material properties when possible [16]. Recent studies using nCeO_2_ coated with aSiO_2_ (amsCeO_2_) have suggested that this approach may prove fruitful, as the coated version of nCeO_2_ was able to substantially reduce nCeO_2_-induced pulmonary inflammation, cytotoxicity, phospholipidosis, and fibrosis following acute exposure in rats [9, 17].

While these studies provide critical insight into the utility of this approach for designing safer ENMs, they were completed primarily using *in vivo* testing models. Given the high number of ENMs in development for countless uses, recently there has been a stronger initiative for more comprehensive *in vitro* models that would allow for more cost- and time-effective screening tools to identify potential toxicities and hazards associated with the use of these ENMs. Consistently, in the current report we sought to validate the reported *in vivo* findings that a nano-thin coating of aSiO_2_ can limit the fibrogenicity of nCeO_2_ [9, 17] using an *in vitro* model consisting of primary normal human lung fibroblast (NHLF) cell cultures. Employing the same nCeO_2_ nanoparticles created using the Harvard Versatile Engineered Nanomaterial Generation System (VENGES), herein we report that nCeO_2_ can directly stimulate NHLFs, inducing both proliferation and excessive collagen production, both of which are classic hallmarks of fibrogenesis. Furthermore, exposure to nCeO_2_ significantly induced the formation of fibrotic nodules in cultured NHLFs. In clinical fibrosis, the presence and number of such fibrotic foci is one of the most reliable indicators of a poor prognosis [18–20]. As anticipated, the nano-thin aSiO_2_ coating limited the fibrogenicity of nCeO_2_, significantly decreasing the extent of nCeO_2_-induced fibroblast proliferation, collagen production, and nodule formation, to levels resembling non-treated control cultures. Collectively these results further validate “safety-by-design” strategies for formulating ENMs and offer the details of a reliable *in vitro* model to analyze fibrogenicity that mimics results observed *in vivo*.

## Results

### Preparation and characterization of particle liquid suspensions for cellular studies

The nano-scaled cerium oxide (nCeO_2_), amorphous silica-coated nCeO_2_ (amsCeO_2_), and the amorphous silica (aSiO_2_) used in these studies were all generated using the Harvard VENGES method. The aSiO_2_ was utilized in these studies as a control particle, since this is the particle with which the nCeO_2_ was encapsulated to create the amsCeO_2_ nanoparticle. In order to further validate our *in vitro* model as it relates to *in vivo* observations, we also included the use of an additional uncoated nCeO_2_ that was not created using the VENGES system, but rather, was purchased commercially from Sigma-Aldrich (denoted as Sigma in the figures) and was tested previously in a rat model in which it was found to induce fibrosis [10, 12]. Single-walled carbon nanotubes (SWCNTs) were also used as a positive control based on their previously reported fibrogenicity [21]. Table 1 summarizes particle characterization data in both powder form and in liquid suspension. Previously published transmission and scanning electron microscopic images of the VENGES nCeO_2_ and amsCeO_2_ samples collected *in situ* illustrate the fractal structure of the agglomerates formed by the flame synthesis method [17]. X-ray diffraction (XRD)-determined crystal size of uncoated nCeO_2_ (17.3 nm) was slightly smaller than that of amsCeO_2_ (21 nm). The specific surface area (SSA) of nCeO_2_ was 61 m^2^/g and the SSA of amsCeO_2_ was 50 m^2^/g, corresponding to an equivalent diameter of 12.8 nm and 19.2 nm, respectively. Differences between the nCeO_2_ and amsCeO_2_ Brunauer–Emmett–Teller (BET)-equivalent diameter are more pronounced than crystal sizes due to the aSiO_2_ encapsulation, which is not accounted for when measuring the crystal size.

**Table 1:** Characterization of the engineered nanoparticles

Nanomaterial	Manufacturer	Manufacturing process	Theoretical aSiO_2_ (wt%)	TCT (nm)	d_XRD_ (nm)	d_BET_ (nm)	SSA (m^2^/g)
nCeO_2_	Harvard University	VENGES FSP	0	0	17.3	12.8	61
amsCeO_2_	Harvard University	VENGES FSP	20	3	21	19.2	50
aSiO_2_	Harvard University	VENGES FSP	100	n/d	n/d	14	195
Sigma CeO_2_	Sigma-Aldrich	n/d	0	n/d	n/d	20	n/d
SWCNT	CNI Houston	HipCo	0	n/d	n/d	n/d	400 – 1200

Abbreviations: *nCeO*_*2*_ uncoated nCeO_2_, *amsCeO*_*2*_ amorphous SiO_2_-coated nCeO_2_, *aSiO*_*2*_ amorphous SiO_2_, *n/d* measurement not determined or data not available, *VENGES FSP* versatile engineered nanomaterial generation system flame spray pyrolysis, *HipCo* high pressure CO disproportionation, *TCT* theoretical coating thickness, *d*_*XRD*_ x-ray diffraction calculated diameter, *d*_*BET*_ Brunauer–Emmett–Teller calculated primary particle diameter, *SSA* specific surface area

Both the intensity- and volume-weighted hydrodynamic diameter (d_H_), as well as the zeta potential of nCeO_2_, amsCeO_2_, and aSiO_2_ dispersed in sterilized deionized water (DI H_2_O) and complete fibroblast growth media (FGM) are summarized in Table 2. The zeta-potentials measured for both aSiO_2_ and amsCeO_2_ dispersed in DI H_2_O were strongly negative and significantly different from the strongly positive zeta potential measured for nCeO_2_. All three particles exhibited similar zeta potential values around −12 mV when dispersed in FGM. Agglomerate diameters as measured via dynamic light scattering (DLS) were stable over the course of 24 h (data not shown). The intensity-weighted d_H_ for nCeO_2_ was 229.7 nm, while that of amsCeO_2_ was 288.3 nm when the particles were suspended in FGM. The polydispersity of both particles were below 0.5, which is indicative of distribution of monodispersed particles. Additionally, the volumetric centrifugation method (VCM)-measured effective density of nCeO_2_ was 1.65 g/cm^3^, whereas that of amsCeO_2_ was 1.54 g/cm^3^ when suspended in FGM.

**Table 2:** Properties of ENM dispersions in DI H_2_O and fibroblast growth medium

Nanomaterial	Media	Intensity-weighted d_H_ (nm)	PdI	Volume-weighted d_H_ (nm)	ζ (mV)	σ (mS/cm)	ρ_agg_ (g/cm^3^)
nCeO_2_	DI H_2_O	262.5 ± 28.56	0.398 ± 0.03	n/d	47.3 ± 6.22	0.0051 ± 0.489	n/d
	FGM	229.7 ± 29.86	0.451 ± 0.11	187.79 ± 16.9	−12.6 ± 2.83	10.7 ± 0.306	1.65
amsCeO_2_	DI H_2_O	222.1 ± 8.86	0.249 ± 0.03	n/d	−50.3 ± 2.00	0.0031 ± 6.08e-5	n/d
	FGM	288.3 ± 5.43	0.354 ± 0.02	278.65 ± 11.8	−13.6 ± 0.92	8.00 ± 0.552	1.541
aSiO_2_	DI H_2_O	132.1 ± 4.45	0.190 ± 0.004	n/d	−36.4 ± 3.81	0.0026 ± 1.57e-4	n/d
	FGM	478.9 ± 35.31	0.437 ± 0.05	685.58 ± 263.9	−11.2 ± 2.18	13.0 ± 1.73	1.075

Abbreviations: *n/d* measurement not determined, *d*_*H*_ hydrodynamic diameter, *PdI* polydispersity index, *ζ* zeta potential, *σ* specific conductance, *ρ*_*agg*_ effective density. Values represent the mean (± SD) of a triplicate reading. Footnote: the average diameters obtained from DLS characterization are derived from the intensity-weighted distributions based on the intensity of light scattered by the particle

**Fig. 1:**
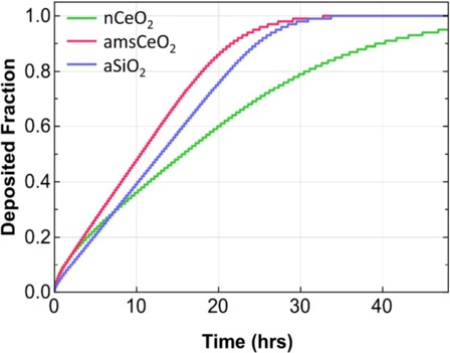
Fraction of deposited nCeO_2_, amsCeO_2,_ and aSiO_2_ particles over a 48 h exposure, using fibroblasts cultured in 24-well tissue culture plates in fibroblast growth medium. The Harvard *in vitro* dosimetry method was used to determine the fraction of nanoparticle delivered to cells as a function of time. Uncoated nCeO_2_ settled slower than both amsCeO_2_ and aSiO_2_, with all nanoparticles achieving approximately 90 % deposition by 40 h post-administration to cells

Both the hydrodynamic diameter and the effective density of these formed agglomerates can greatly affect the fate and transport of the particles *in vitro*, in addition to defining settling rates, with significant implications for the dose of particles actually delivered to cells over the course of the *in vitro* toxicity assay. Thus, the delivered dose of nanoparticles may not always equal the administered dose [22–24]. In an effort to calculate the delivered-to-cell dose of nCeO_2_, amsCeO_2_, and aSiO_2_, we employed the recently developed Harvard *in vitro* dosimetry method [22], which determines the fraction of the administered particles that is deposited onto the cells in the bottom of the well as a function of time (Table 3 and Fig. 1). As anticipated, the nanoparticles did not behave exactly the same in the FGM, with the nCeO_2_ settling at a slower rate than both the amsCeO_2_ and aSiO_2_. While the amsCeO_2_ and aSiO_2_ achieved nearly 100 % deposition of the administered dose by approximately 24 h, roughly 70 % of the administered dose of nCeO_2_ was delivered to the cells over the same time frame. nCeO_2_ did not reach the same level of deposition as amsCeO_2_ or aSiO_2_ until at least 40 h post-treatment. Based on these results, the analyses reported herein were carried out 48 h post-exposure to the nanoparticles to allow for complete deposition of all particles.

**Table 3:** Relevant *in vitro* dose (RID) functions for nCeO_2,_ amsCeO_2,_ and aSiO_2_ nanoparticles when dispersed in FGM

Nanomaterial	M	N	SA	RID_M_	RID_N_	RID_SA_
nCeO_2_ Low dose	0.01	2.08E-09	3.63E-16	0.01	1.08E-16	1.88E-23
nCeO_2_ High dose	0.40	4.25E-01	4.53E-01	0.36	2.20E-08	2.35E-08
amsCeO_2_ Low dose	0.01	6.82E-10	3.90E-17	0.01	5.88E-17	3.37E-24
amsCeO_2_ High dose	0.40	3.10E-01	2.41E-01	0.39	2.68E-08	2.08E-08
aSiO_2_ Low dose	0.01	6.56E-11	9.69E-05	0.01	3.11E-18	4.60E-12
aSiO_2_ High dose	0.40	1.10E + 00	1.62E + 06	0.39	5.20E-08	7.68E-02

Delivered dose values based on delivered ENM mass (M), delivered particle number (N), and delivered surface area (SA) for either a high (0.57 μg/mL, equivalent to 0.2 μg/cm^2^ for this study) or low (0.017 μg/mL, 0.006 μg/cm^2^) dose

Interestingly, the observed fibrogenic effects of nCeO_2_ following a 24 h exposure did reveal a similar trend as the results presented herein that were obtained at the 48 h timepoint (data not shown). Although these results did not reach statistical significance at 24 h post-treatment, these findings highlight the increased fibrogenic potential of the nCeO_2_ compared to the amsCeO_2_, since there was roughly 30 % less deposition of the nCeO_2_ at that time.

### nCeO_2_ induces proliferation and collagen I expression in fibroblasts

As previously mentioned, nCeO_2_ is known to accumulate in the lungs in both humans and in animal models, and has been found in the pulmonary interstitium long after exposure has ceased [1, 6, 10]. This would suggest that nCeO_2_ would directly contact resident lung cell types, including fibroblasts. In an effort to determine whether nCeO_2_ directly stimulates fibroblasts, we measured two hallmarks of fibrosis, cell proliferation and collagen production. Primary human fibroblasts were treated with a low dose, 0.006 μg/cm^2^, as well as a high dose, 0.2 μg/cm^2^, which correspond to the physiologically relevant doses that induced pulmonary fibrosis in a rat model [10, 12]. In these *in vivo* studies, the lowest dose of nCeO_2_ tested was 0.15 mg/kg body weight. With an average weight of approximately 200 g per rat as noted in the report, this dose translates to approximately 30 μg per animal. Based on the rat lung surface area of 4000 cm^2^, this corresponds to a dose of approximately 0.0075 μg/cm^2^, which was sufficient to induce fibrosis at 84 days post-treatment. Using the same calculations, the 3.5 mg/kg dose corresponds to approximately 0.2 μg/cm^2^, and this dose induced fibrosis 28 days post-treatment [10, 12].

**Fig. 2:**
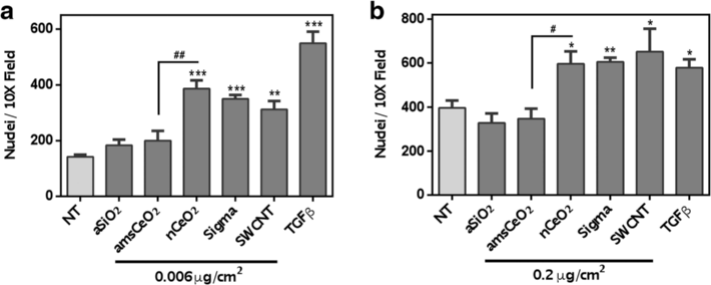
nCeO_2_ directly induces proliferation of NHLFs, while amsCeO_2_ does not. Quantitation of DAPI stained nuclei of NHLFs that had been treated for 48 h was used to determine cell counts at the indicated doses and treatments. nCeO_2_ induced a significant increase in cell proliferation at both 0.006 μg/cm^2^ (**a**) and 0.2 μg/cm^2^ (**b**) compared to non-treated cells as well as those treated with amsCeO_2_. SWCNTs and TGFβ (1 ng/mL) were used as positive controls and also significantly increased proliferation of the fibroblast cells. Statistical significance is indicated as *p < 0.05, **p < 0.01, and ***p < 0.001 compared to non-treated (NT) conditions and #*p* < 0.05 and ##*p* < 0.01 for nCeO_2_ compared to amsCeO_2_

Following treatment with both the low and high doses, cell proliferation was analyzed via nuclear stain and subsequent quantitation, which revealed that nCeO_2_ significantly increased NHLF cell proliferation compared to non-treated cells (Fig. 2). In contrast, amsCeO_2_ did not induce cell proliferation at either dose, similar to the aSiO_2_ control, and treatment with the amsCeO_2_ resulted in proliferation levels that were significantly lower than the nCeO_2_ (Fig. 2). As mentioned, in order to further validate our *in vitro* model as it relates to *in vivo* observations, we also included the use of an additional uncoated nCeO_2,_ denoted as Sigma in the figures, which also induced cell proliferation at both doses tested, similar to the uncoated nCeO_2_ obtained using the VENGES method.

Previous studies from our group have demonstrated that SWCNTs are directly fibrogenic to fibroblast cells, inducing cell proliferation as well as collagen production [21]. Thus, these particles were used as a positive control in the studies reported herein. As expected, SWCNTs also induced cell proliferation at levels similar to both uncoated nCeO_2_ particles (Fig. 2). Similarly, the known fibrosis-inducing cytokine transforming growth factor beta (TGFβ) was used as a positive control and, as anticipated, invoked a significant level of proliferation in these cells.

**Fig. 3:**
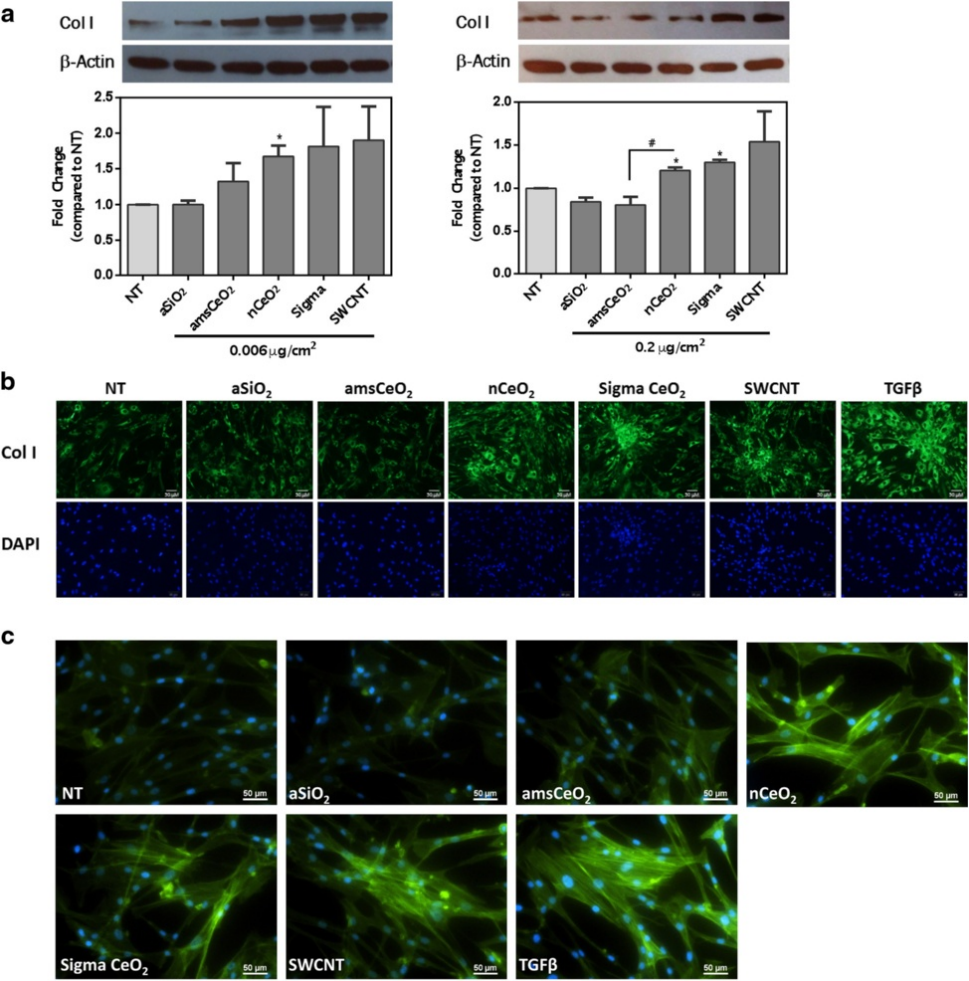
nCeO_2_ induces collagen I production from NHLFs, a hallmark of fibrosis, while amsCeO_2_ does not. Primary human lung fibroblasts were treated with the indicated nanoparticle for 48 h. Western blot analysis with corresponding densitometry (**a**) and fluorescence microscopy (**b**) revealed a nCeO_2_-induced increase in collagen I production, while the amorphous silica coating of nCeO_2_ attenuated this observed effect on collagen production. SWCNTs and TGFβ (1 ng/mL) also significantly increased the expression of collagen I, as expected. (**c**) Alpha-smooth muscle actin staining of NHLFs that had been treated for 48 h with 0.2 μg/cm^2^ of the indicated nanoparticle demonstrated that nCeO_2_, as well as the Sigma CeO_2_, SWCNTs, and TGFβ, increased expression of this myofibroblast marker. In contrast, the amsCeO_2_, as well as the aSiO_2_ control, expressed levels consistent with non-treated (NT) cells. Statistical significance is indicated as *p < 0.05 compared to NT conditions and #*p* < 0.05 for nCeO_2_ compared to amsCeO_2_

As discussed, fibrosis is associated with the activation of fibroblast cells that stimulates the release and accumulation of extracellular matrix proteins such as collagen. Hence, collagen production was measured via Western blot analysis, as well as fluorescent microscopy, to determine whether nCeO_2_ can induce the release of collagen directly from fibroblast cells. As demonstrated in Fig. 3, we observed a significant increase in collagen production in response to both uncoated nCeO_2_ particles, as well as both positive controls, SWCNT and TGFβ, compared to non-treated cells. This effect was not observed in cells that had been treated with amsCeO_2_. Furthermore, fibroblast cells treated with 0.2 μg/cm^2^ of nCeO_2_, as well as the Sigma CeO_2_, SWCNTs, and TGFβ, demonstrated increased expression of alpha-smooth muscle actin (α-SMA), which indicates the activation and subsequent transformation of fibroblasts into myofibroblasts (Fig. 3c), the main source of collagen production during wound healing and fibrotic events. In contrast, the amsCeO_2_, as well as the aSiO_2_ alone, expressed α-SMA at levels similar to the non-treated cells. Taken together with the cell proliferation data, these results demonstrate that the aSiO_2_ encapsulation of nCeO_2_ is capable of attenuating the fibrogenic nature of the nCeO_2_ as measured *in vitro*, in agreement with *in vivo* reports [9].

### nCeO_2_, but not amsCeO_2_, induces the formation of fibroblastic nodules

**Fig. 4:**
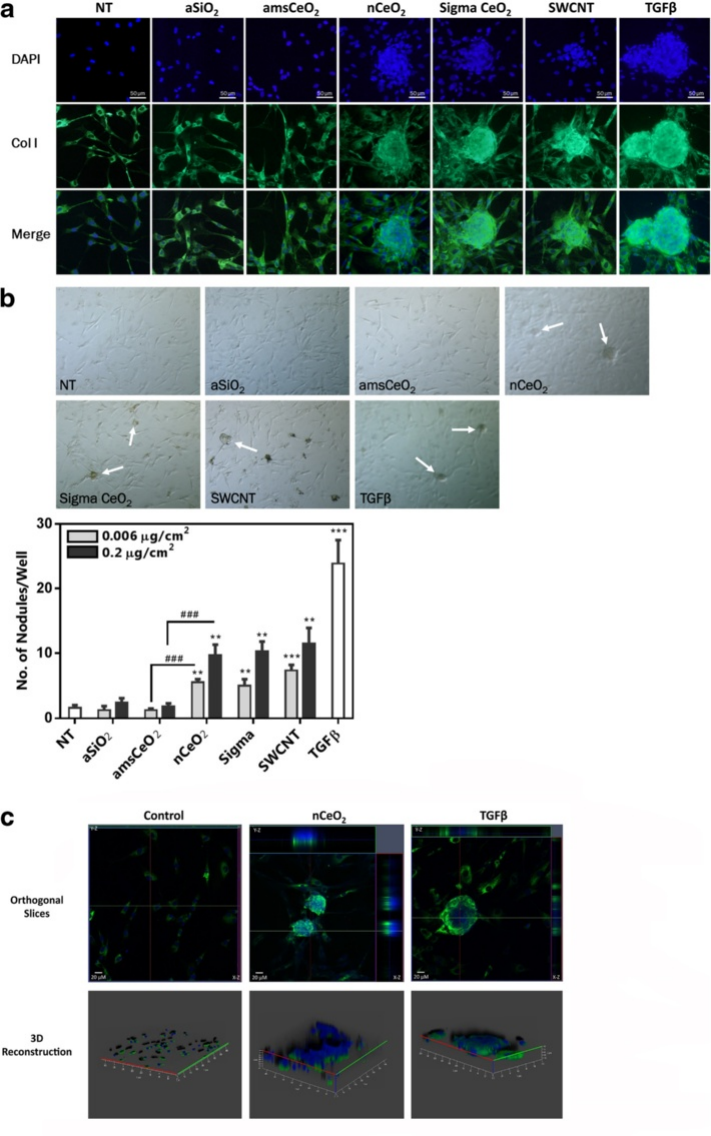
nCeO_2_, but not amsCeO_2_, directly induces the formation of fibroblastic nodules in NHLFs in a dose dependent manner. Fibroblasts were plated on poly-L-lysine coated coverslips and treated with the indicated nanoparticle at 0.006 or 0.2 μg/cm^2^ for 48 h. Cells were then stained for collagen I and DAPI and fibroblastic nodule formation was determined for each condition as the number of nodules formed per well. (**a**) Representative images of nodule formation in response to 0.2 μg/cm^2^ of the indicated particles. Collagen I expression is shown in green and nuclear stain (DAPI) in blue. (**b**) Brightfield images of the fibroblastic nodules (arrows) were used to quantitate the number of nodules formed per well in response to each of the indicated nanoparticles. Statistical significance is indicated as *p < 0.05, **p < 0.01, and ***p < 0.001 compared to non-treated (NT) conditions and ###*p* < 0.001 for nCeO_2_ compared to amsCeO_2_. (**c**) Confocal microscopy Z-stack analysis with orthogonal views (upper panels) of the X-Z plane (right) and Y-Z plane (top). Three dimensional reconstruction was generated (bottom panels) based on the results of the Z-stack images and demonstrated increased thickness of fibrotic nodule clusters formed in response to nCeO_2_ and TGFβ as compared to the untreated monolayer of NHLFs, indicating the mounding of the cells within the nodule

We next utilized a fibroblastic nodule assay to further analyze the fibrogenicity of nCeO_2_, as well as the effect that the encapsulation of this particle with aSiO_2_ has on this process. A key pathological characteristic of fibrosis is the formation of fibrotic foci composed of fibroblasts, myofibroblasts, and accumulated collagen [18, 25, 26]. Consistently, formation of these foci *in vitro* is recognized as a useful method to assess the fibrogenic potential of nanomaterials. Primary human lung fibroblasts were plated on coverslips that had been coated with poly-L-lysine and subsequently treated with nanoparticles, as indicated, and nodule formation was assessed. As anticipated, we observed that both of the uncoated forms of nCeO_2_ directly and significantly induced the formation of fibroblastic nodules in these cells in a dose dependent manner, as did both positive controls (Fig. 4). The amsCeO_2_ did not induce extensive formation of fibroblastic foci, stimulating the formation of only a few foci per well, consistent with numbers observed in the non-treated cell cultures, and significantly less than nCeO_2_. In addition, confocal microscopy z-stack analysis demonstrated that the fibrotic nodule clusters formed in response to nCeO_2_ and TGFβ had an increased thickness as compared to the untreated monolayer of NHLFs, indicating the formation of multi-layer nodules (Fig. 4c).

**Fig. 5:**
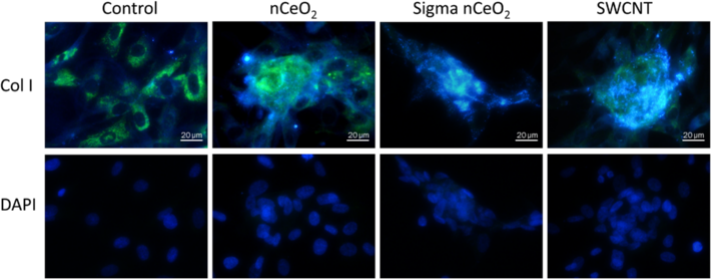
nCeO_2_ particles localize within fibroblastic nodules. NHLFs were treated with 0.2 μg/cm^2^ of the indicated nanoparticle for 48 h. Following the treatments, cells were fixed, stained, and mounted onto glass, laser cut slides. Enhanced darkfield microscopy using the CytoViva illumination system revealed that the nanoparticles (bright spots) are localized on and around the fibroblastic nodules, suggesting that the accumulated particles may play a role in the initiation or formation of the nodules

Further analysis of the fibrotic nodules using enhanced darkfield imaging revealed that the nCeO_2_ localizes at the site of nodule formation (Fig. 5). Consistently, SWCNTs also localized with the resulting fibrotic foci. Given that nCeO_2_ has been shown to accumulate in the lungs following exposure [1, 6, 10, 27–29], this data would suggest that the formation of nodules may occur, at least in part, due to the accumulation of aggregated nanoparticles on the fibroblast cells that then respond to the foreign material in an aberrant manner.

### The fibrogenic effect of nCeO_2_ is mediated by TGFβ signaling

**Fig. 6:**
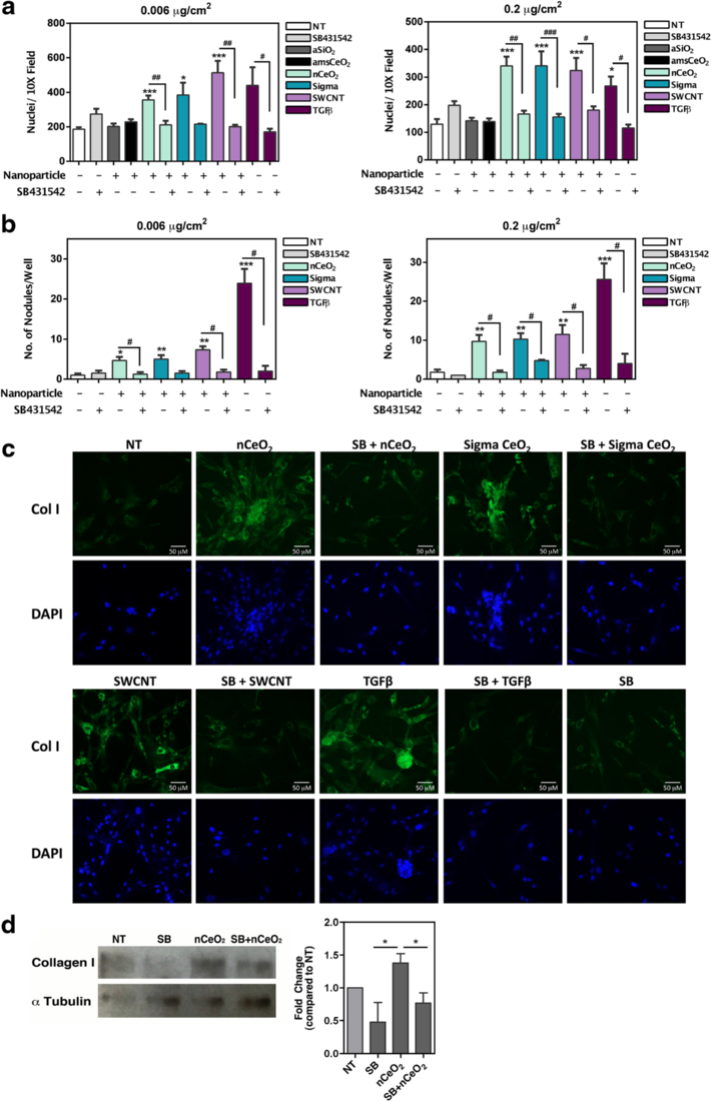
nCeO_2_ induced cell proliferation and fibroblastic nodule formation are attenuated with chemical inhibition of TGFβ receptor signaling. NHLFs were treated as indicated for 48 h. Cells receiving the TGFβ receptor inhibitor SB431542 (SB) were treated for 3 h with 1 μM of inhibitor prior to the addition of the nanoparticles. Chemical inhibition of the TGFβ receptor reversed the nCeO_2_-induced increase in cell proliferation (**a**) and fibroblastic nodule formation (**b**) at both of the tested nanoparticle doses. (**c**) Collagen I staining demonstrated that inhibition of the TGFβ receptor decreased the nCeO_2_-induced increase in collagen I production from NHLFs. (**d**) Western blot analysis and corresponding densitometry further confirmed that the SB inhibitor reversed the nCeO_2_-induced increase in collagen I production. Statistical significance is indicated as *p < 0.05, **p < 0.01, and ***p < 0.001 compared to non-treated (NT) conditions or compared to other treatments as indicated in D; #*p* < 0.05, ##*p* < 0.01, and ###*p* < 0.001 compared to the same treatment condition without inhibitor

Finally, we sought to determine whether TGFβ signaling was the mechanism by which nCeO_2_ was inducing stimulation of the fibroblast cells, since this mediator has been found to be upregulated following pulmonary instillation of nCeO_2_ in rats [10], and is a known pro-fibrotic signal. As such, the TGFβ receptor inhibitor SB431542 was used, and fibroblast cells were incubated with this inhibitor for 3 h prior to the addition of nanoparticles. Following suppression of TGFβ signaling, nCeO_2_-induced cell proliferation was diminished to levels resembling controls (Fig. 6a). Similarly, analysis of fibrotic nodule formation revealed that inhibition of TGFβ signaling attenuated nCeO_2_-induced fibrogenicity (Fig. 6b). Inhibition of TGFβ signaling also decreased collagen I production as induced by nCeO_2_, demonstrated by decreased collagen I staining of NHLFs (Fig. 6c) and via western blot analysis (Fig. 6d). Collectively, these results indicate that nCeO_2_ induces TGFβ signaling in fibroblast cells that ultimately leads to increased fibrogenicity.

## Discussion

Use of nCeO_2_ is quickly on the rise worldwide, with an increasing number of applications. Additionally, the increased use as a diesel fuel additive is leading to elevated levels of nCeO_2_ released in diesel fuel exhaust, further increasing exposure to the general public [5]. Currently, reports of the implications of nCeO_2_ treatment, both in cell culture models as well as animal models, tend to be conflicting in their determination of whether nCeO_2_ is hazardous or safe. Indeed, some report the potential efficacy of this compound as a therapeutic agent based on its anti-oxidant and anti-inflammatory properties [30–32]. Still others, such as those discussed within this report, contend that nCeO_2_ is a potential hazard due to its observed fibrogenicity. This discrepancy seems to be based primarily on the route of exposure, with inhalation being the most hazardous. These results, taken together with reports of nCeO_2_-induced pulmonary disease and fibrosis following inhalation exposure [5, 6, 10, 12], raise public health concerns due to the fact that most current applications of nCeO_2_ employed have the ability to generate respirable particles.

The potential health hazards caused by exposure to ENMs such as nCeO_2_ has led to the development of alternative engineering methods to create safer materials while still maintaining the desirable characteristics of the material, commonly referred to as “safety-by-design” strategies. One such method employs a nano-thin layer of amorphous silica to “mask” the outside of the particle, since amorphous silica is considered to be relatively biologically inert [16, 33]. One main concern with these types of strategies is that the core material will no longer possess its desired properties once encapsulated. Indeed, in the case of CeO_2_, the desirable catalytic properties may not be retained with an aSiO_2_ coating; thus, depending on the application, this strategy may not always prove fruitful. However, when considering the observed results obtained in rat models, that amsCeO_2_ causes less acute inflammation and pulmonary cell toxicity as compared to the uncoated nCeO_2_, and minimizes markers of fibrosis such as the accumulation of surfactant in the air space and collagen production [9, 17], it seems that this strategy is worth pursuing when the application would tolerate such coatings. Consistent with this notion, nano-thin aSiO_2_ coating also demonstrated a protective effect when used to encapsulate iron oxide, reducing both toxicity and genotoxic effects in two different cell types [34]. Collectively these reports, as well as the results demonstrated herein, validate this concept of “safety-by-design” for ENMs for further study and development.

As previously mentioned, current reports focused on nCeO_2_ provide conflicting notions regarding the safety or potential toxicity of this nanoparticle. While some of these discrepancies can be attributed to the method of exposure, such as ingestion versus inhalation, others may be more attributable to differences in the toxicological experimental methods employed (e.g., dosing, timing, dispersion method, measured outcome). Unfortunately, there is limited data on actual human exposure concentrations to nCeO_2_, and this makes predicting physiologically relevant doses somewhat difficult for experimental investigation. Likewise, nanoparticles are known to behave differently in various types of cell culture or dispersion media, often due to effects like the formation of protein coronas which can alter the bio-interactions of these particles once delivered to cells [17, 35, 36]. Several *in vitro* studies have examined the potential toxicity of nCeO_2_, using numerous cell lines and endpoints, and most have concluded that nCeO_2_ is relatively non-toxic and does not illicit a strong inflammatory response [17, 37–40]. On the contrary, when investigated *in vivo*, nCeO_2_ has demonstrated both pro-inflammatory and pro-fibrogenic properties [8–10, 12, 14, 17, 41], which seems more consistent with the reported health effects observed in human case studies [6].

These apparent disparities between *in vitro* and *in vivo* results make evaluating the potential hazards of nCeO_2_ difficult, and highlight the need for further development of more reliable and consistent *in vitro* models to study and predict the fibrogenicity potential of ENMs. This is especially important when considering the fact that animal studies can be time-consuming, costly, and limited in terms of high-throughput potential when screening a large number and variety of particles. Given the vast number of ENMs in use and in development, a number that continues to grow daily, this notion seems more critical than ever. Furthermore, *in vitro* cell models are useful in allowing more in depth analysis of the specific mechanisms employed by certain cell types as a result of exposure that cannot be assessed *in vivo* due to the complexity of the entire system. These mechanistic details can provide vital evidence that may be utilized for the purpose of developing therapeutic and diagnostic strategies or outcomes. For example, our model allowed us to utilize the TGFβ inhibitor SB431542 to investigate the extent to which TGFβ signaling plays a role in nCeO_2_-induced fibrogenicity specifically in fibroblast cells, which we would not have been able to accomplish *in vivo* (Fig. 6). *In vivo* models will always remain a necessary approach to predict human response and potential disease outcomes, however, development of more novel, reliable *in vitro* methods would be extremely advantageous in helping to identify select agents that should be fully characterized using *in vivo* approaches.

In light of these concerns, we sought to develop a reliable *in vitro* model to examine the potential fibrogenicity of nCeO_2_ using primary human fibroblasts. These cells present a desirable cell type for *in vitro* analysis of nCeO_2_ since this particle is known to persist in the lung and has been found in the interstitium [1, 6, 10, 27–29]. Furthermore, *in vivo* models suggest that the primary negative outcome of exposure to nCeO_2_ is fibrosis, which lends to the notion that fibroblast cells may be the main cell type involved in the biological response to nCeO_2_. Thus, we directly treated primary human fibroblasts with nCeO_2_ or amsCeO_2_ to assess fibrogenicity. Our group has also previously employed this strategy to evaluate nanomaterials, specifically carbon nanotubes, and found that this model consistently correlated with *in vivo* outcomes [21, 42]. In the current report, we observed increased markers of fibrogenicity, cell proliferation, and collagen production (Figs. 2 and 3) when NHLFs were directly treated with nCeO_2_ at physiologically relevant doses that match those reported *in vivo* [9, 10, 12]. nCeO_2_ is relatively insoluble under typical cell culture conditions [43], indicating that the effects reported here are likely due to cellular interactions with particulate nCeO_2_ rather than dissolved cerium. In addition, we saw a significant increase in the formation of fibrotic foci when the cells were treated with nCeO_2_ (Fig. 4). These findings demonstrate that this *in vitro* model was able to accurately mimic results obtained using *in vivo* models [9, 10, 12, 17]. Similarly, our results accurately portrayed the observed *in vivo* effects of the nano-thin amorphous silica coating of nCeO_2_ when exposed to the NHLF cells, showing cell proliferation, collagen expression, and fibrotic nodule formation at levels consistent with non-treated and negative controls. These results also further validate the “safety-by-design” approach for further development of ENMs.

As mentioned, the higher dose of nCeO_2_, 0.2 μg/cm^2^, employed in our studies is equivalent, based on body weight and surface area of the rat lung, to the physiologically relevant dose that has been used in numerous *in vivo* models to initiate fibrosis. Indeed, using this dose we also observed the correlative fibrogenic potential of nCeO_2_ in our *in vitro* model. Interestingly, the low dose of 0.006 μg/cm^2^ used in these studies, which scarcely induces fibrosis *in vivo*, also induced a significant fibrogenic effect in our model. These results are noteworthy since they indicate that even very low levels of exposure may have the potential to initiate a fibrotic response. This is especially important in the case of nanomaterials when considering their bio-persistence. In addition, this finding highlights the notion that reliable *in vitro* models may be useful in identifying potentially harmful doses of materials that would be relevant for follow up using *in vivo* models. Indeed, it plausible that interstitial fibroblasts would be exposed to lower doses of inhaled nanoparticles than other cell types of the lungs following exposure, due to factors such as clearance as well as translocation rate into the interstitium.

## Conclusions

In summary, the results presented herein demonstrate that treatment of primary human lung fibroblasts *in vitro* is a useful approach for evaluating the potential fibrogenicity of nanomaterials that can efficiently represent results obtained *in vivo*. Furthermore, our results confirm those obtained using an *in vivo* rat model that a nano-thin coating of amorphous silica on the surface of nCeO_2_ can successfully ameliorate the fibrogenicity of this nanomaterial. These results not only support the notion that “safety-by-design” strategies for developing ENMs are useful, but also demonstrate the utility of the current *in vitro* model for testing the fibrogenic potential of respirable nanoparticles.

## Methods

### Reagents and antibodies

Fibroblast basal medium, growth supplements, and NHLFs were purchased from Lonza (Walkersville, MD). Human-derived transforming growth factor beta (TGFβ), as well as the TGFβ receptor blocker SB431542, was obtained from R&D Systems, Inc. (Minneapolis, MN). Poly-L-lysine was purchased from Sigma-Aldrich (St. Louis, MO). The anti-Collagen I antibody was purchased from Fitzgerald (Acton, MA), while the alpha tubulin antibody was purchased from Santa Cruz Biotechnology, Inc. (Dallas, TX). Alexa-fluor 488-conjugated donkey anti-rabbit secondary antibody, alexa-fluor 488-conjugated alpha-smooth muscle actin (α-SMA) primary antibody, and the beta actin primary antibody were purchased from Abcam (Cambridge, MA). ProLong gold antifade with DAPI mountant was obtained from Molecular Probes (Thermo Fisher Scientific, Waltham, MA).

### Particle generation and characterization

Nano-scaled CeO_2_, amsCeO_2_, and aSiO_2_ particles were generated using the Harvard Versatile Engineered Nanomaterial Generation System (VENGES) and characterized as previously described [17]. Briefly, x-ray diffraction (XRD) patterns were obtained using a Scintag XDS2000 powder diffractometer [Cu Kα (λ = 0.154 nm), −40 kV, 40 mA, stepsize = 0.02°]. The crystal size was determined by applying the Sherrer Shape Equation to the Gaussian fit of the major diffraction peak. The Brunauer–Emmett–Teller (BET) powder-specific surface area (SSA) of all samples was measured by nitrogen adsorption at 77 K (Micromeritics TriStar; Norcross, GA), after sample degassing for 1 h at 150 °C in nitrogen. BET equivalent primary particle size was calculated, under a spherical particle assumption, using d_BET_ = 6000/(ρ × SSA), where ρ is the material density.

Dry nCeO_2_, amsCeO_2_, and aSiO_2_ particle dispersions in complete fibroblast growth medium (FGM) were prepared using a protocol developed by the authors [23], in which the particle-specific critical delivered sonication energy (DSE_cr_), hydrodynamic diameter (d_H_), formed agglomerate size distribution, polydispersity index (PdI), zeta potential (ζ), specific conductance (σ), pH, colloidal stability, and effective density of formed agglomerates are measured [44]. For treatment of cell cultures, particles were suspended in DI H_2_O to acquire stock solutions of 0.1 mg/mL. Immediately prior to use, all particles were dispersed via sonication and subsequently diluted in FGM to the desired concentrations. Particles were then briefly vortexed and slowly added to the cultures.

### Dosimetry

Subsequent to particle suspension, preparation, and characterization, the delivered-to-cell mass was calculated. Unlike soluble chemicals, colloidal suspensions of nanoparticles can have altered surface area, agglomerate formation, and settling rates depending on the suspension media, which may impact the bio-interactions of the particles when deposited onto cells in culture. Thus, dosimetric considerations must be taken into account. Therefore, the fraction of administered particle mass that is deposited on the cells as a function of *in vitro* exposure time (f_D_) was calculated [22, 44] in order to accurately replicate the *in vivo* lung deposited doses of nCeO_2_ [9, 10, 12]. The f_D_ as a function of *in vitro* exposure time was calculated using the hybrid Volumetric Centrifugation Method-*In Vivo* Sedimentation, Diffusion and Dosimetry (VCM-ISDD) methodology [22, 44, 45] developed by the authors. The mean media-formed volume-weighted agglomerate hydrodynamic diameter (d_H_) and the VCM-measured effective density of formed agglomerates [44] were used as inputs to the VCM-ISDD fate and transport numerical model in order to estimate the f_D_ as a function of time.

### Preparation of carbon nanotubes

Single-walled carbon nanotubes (SWCNT; Carbon Nanotechnologies, Inc., Houston, TX) were produced by the high pressure CO disproportionation (HipCo) technique, using CO in a continuous-flow gas phase as the carbon feedstock and Fe (CO)_5_ as the iron-containing catalyst precursor. The SWCNTs were subsequently purified by acid treatment to remove metal contaminants. Nitric acid dissolution and inductively coupled plasma-atomic emission spectrometry (ICP-AES, NMAM #7300) were used to perform an elemental analysis of the supplied SWCNTs, and indicated that they contained 99 % elemental carbon and 0.23 % iron. The specific surface area was measured at −196 °C by the nitrogen absorption-desorption technique (BET) using a SA3100 Surface Area and Pore Size Analyzer (Beckman Coulter, Fullerton, CA). The surface area of dry SWCNT was 400 – 1,000 m^2^/g, and the length and width of individual (dry) SWCNT was 0.1 – 1 μm and 0.8 – 1.2 nm, respectively.

Dry SWCNTs were prepared for treatment as previously described and validated [46]. Briefly, particles were suspended in distilled water to acquire stock solutions of 0.1 mg/mL, which were supplemented subsequently with 150 μg/mL of a natural lung surfactant, Survanta (Abbott Laboratories, Columbus, OH) to aid in their dispersion. All particles were then dispersed using brief sonication and diluted in culture medium to the desired concentration.

### Cell culture

NHLFs were obtained from Lonza (Walkersville, MD) and cultured in FGM, which is composed of Fibroblast Cell Basal Medium (FBM; Lonza) that contains all recommended growth supplements (FGM™-2 BulletKit™; Lonza). Cells were cultured and maintained at sub-confluent densities in a humidified incubator at 37 °C with 5 % CO_2_. For each experiment, cells were seeded at density of 2.5 ×10^4^ cells per well in a 24-well plate the day before treatments were performed.

### Immunofluorescence

Cells were seeded at a density of 2.5 × 10^4^ cells per well onto glass coverslips that had been treated with poly-L-lysine (0.1 μg/mL), rinsed, and dried. Prior to their addition to cells, stock solutions of nanoparticles were sonicated and subsequently diluted in FGM to the desired concentration. Treated fibroblasts were incubated with nanoparticles for 48 h and were then fixed in 4 % paraformaldehyde and permeabilized using phosphate buffered saline containing 0.25 % Triton-X 100. Cells were then blocked with 2 % normal donkey serum and incubated with primary antibody raised against collagen I, followed by fluorescently conjugated secondary antibody. Cover glasses were then mounted on glass slides using ProLong Gold Antifade mountant with DAPI. Fluorescent and brightfield images were captured using a Zeiss Axiovert 100 TV inverted microscope (Carl Zeiss Microscopy, LLC, Thornwood, NY). For CytoViva images, coverslips were mounted onto laser-cut glass slides and images were captured using an Olympus BX-51 Microscope equipped with the CytoViva Advanced Dark field Illumination System (CytoViva, Auburn, AL) and a 100 W quartz-halogen light source.

Alternatively, cells stained for α-SMA were plated into chamber slides (Nalgene Nunc International, Naperville, IL) at a density of 1 ×10^4^ cells per well. Stock solutions of nanoparticles were sonicated and subsequently diluted in FGM to the desired concentration for cell treatment. Fibroblasts were incubated with nanoparticles for 48 h and cells were then fixed with 4 % paraformaldehyde in PBS solution on ice for 30 min and permeabilized with 2 % Triton-X 100 for 30 min at room temperature. After washing, cells were blocked with normal rabbit serum for 1 h and then incubated overnight at 4 °C with primary antibody raised against α-SMA that was conjugated with alexa-fluor 488. Cells were then washed and mounted with mounting solution containing DAPI (Vector Laboratories, Inc., Burlingame, CA). Images were taken using a Nikon Ti Eclipse fluorescent microscope.

### Cell proliferation determination

Fibroblast cells were prepared, treated, and imaged as described for immunofluorescence. Resultant images were then analyzed for total cell numbers via Image J software (Bethesda, MD) by counting the total number of DAPI stained nuclei per 10X field. Four fields were captured per coverslip in duplicate for each of three independent replicates.

### Immunoblot analysis and collagen I quantification

Following treatment, whole cell lysates were collected using cell extraction buffer (Life Technologies, Thermo Fisher Scientific, Waltham, MA) supplemented with 0.1 mM PMSF and a mixture of protease and phosphatase inhibitors. Cellular debris was removed by high-speed centrifugation and protein concentrations were then determined in each sample using the Pierce BCA Protein Assay (Life Technologies, Thermo Fisher Scientific, Waltham, MA). Samples (25 μg protein/lane) were separated on sodium dodecyl sulfate (SDS)-polyacrylamide gels and electrophoretically transferred to nitrocellulose membranes. The membranes were then analyzed with collagen I, beta actin, or alpha tubulin primary antibodies, and bound antibodies were detected using HRP-conjugated secondary antibodies. Densitometry was performed on the resulting bands using Image J software (NIH, Bethesda, MD) to quantitate the protein concentration in each sample.

### Fibroblastic nodule assay

NHLFs were seeded onto glass coverslips that had been treated with poly-L-lysine (0.1 μg/mL) at a density of 2.5 ×10^4^ cells per well in a 24 well plate, and allowed to adhere overnight. Prior to their addition to cells, nanoparticles were sonicated using a cup sonicator and were diluted in FGM to the desired concentration. For experiments employing the TGFβ inhibitor SB431542, cells were incubated with 1 μM of the inhibitor for 3 h prior to addition of the nanoparticles. Fibroblasts were then incubated with nanoparticles for 48 h and were subsequently fixed in 4 % paraformaldehyde and permeabilized using phosphate buffered saline containing 0.25 % Triton-X 100. Cells were then blocked with 2 % normal donkey serum and incubated with primary antibody raised against collagen I, followed by fluorescently conjugated secondary antibody. Cover glasses were then slowly washed and subsequently mounted on glass slides using ProLong Gold Antifade mountant with DAPI. Fluorescent and brightfield Images were captured using a Zeiss Axiovert 100 TV inverted microscope (Carl Zeiss Microscopy, LLC, Thornwood, NY). Confocal microscopy z-stack images were obtained using a Zeiss LSM780 confocal microscope. For each culture, two cover slides were analyzed and counted for nodule formation and data was pooled from three independent replicates.

### Statistical analysis

Data represents mean ± SEM from three or more independent replicates and samples were analyzed using a one-way ANOVA with Tukey’s post-test for multiple comparisons to determine significance. Statistical significance is indicated in each figure as *p < 0.05, **p < 0.01, and ***p < 0.001. All statistical analyses were performed using GraphPad Prism Software (La Jolla, CA).
